# Comparing Corneal Biomechanic Changes between Solo Cataract Surgery and Microhook Ab Interno Trabeculotomy

**DOI:** 10.3390/jcm13154564

**Published:** 2024-08-05

**Authors:** Ryo Asaoka, Shuichiro Aoki, Yuri Fujino, Shunsuke Nakakura, Hiroshi Murata, Yoshiaki Kiuchi

**Affiliations:** 1 Department of Ophthalmology, Seirei Hamamatsu General Hospital, Hamamatsu 430-0906, Shizuoka, Japan; 2 Seirei Christopher University, Hamamatsu 433-8558, Shizuoka, Japan; 3 The Graduate School for the Creation of New Photonics Industries, Hamamatsu 431-1202, Shizuoka, Japan; 4 Department of Ophthalmology, The University of Tokyo Graduate School of Medicine, Tokyo 113-0033, Japan; 5 Department of Ophthalmology, Shimane University Faculty of Medicine, Izumo 693-0021, Shimane, Japan; 6 Department of Ophthalmology, Tsukazaki Memorial Hospital, Himeji 671-1227, Hyogo, Japan; 7 Department of Ophthalmology, National Center for Global Health and Medicine, Shinjuku 162-8655, Tokyo, Japan; 8 Department of Ophthalmology and Visual Science, Hiroshima University, Hiroshima 739-0046, Hiroshima, Japan

**Keywords:** cataract, trabeculotomy, glaucoma, Corvis ST, Ocular Response Analyzer

## Abstract

**Background/Objectives**: This study aimed to examine the postoperative changes in the corneal biomechanical properties between solo cataract surgery and solo microhook ab interno trabeculotomy (LOT). **Methods**: This retrospective case–control study included 37 eyes belonging to 26 patients who underwent solo cataract surgery and 37 eyes belonging to 31 patients who underwent solo µLOT. These two groups were matched according to their preoperative intraocular pressure (IOP), axial length (AL), and age. Corneal Visualization Scheimpflug Technology (Corvis ST) was used to obtain four biomechanical parameters representing the corneal stiffness or corneal deformation at the highest concavity, including stiffness parameter A1 (SP-A1), stress–strain index (SSI), peak distance (PD), and deflection amplitude max (DefAmpMax). These parameters were compared preoperatively and 6 months postoperatively, and between the two surgical groups. **Results**: Preoperatively, the patients’ IOP, age, and AL, as well as their results in four Corvis ST parameters, were similar between the two groups (*p* > 0.05). No significant difference was observed in SP-A1; however, PD and DefAmpMax were significantly larger, and SSI was significantly smaller postoperatively in the LOT group than in the cataract group. **Conclusions**: Corneal stiffness was reduced, and the cornea was more deformed with LOT than cataract surgery.

## 1. Introduction

Glaucoma is one of the leading causes of irreversible blindness in the world. Intraocular pressure (IOP) is the only established modifiable factor for halting the progression of glaucoma. Currently, minimally invasive glaucoma surgery (MIGS) has greatly expanded the surgical treatment options in glaucoma to reduce IOP. Cataract surgery is often performed in aged patients, and many studies have reported a decrease in IOP after cataract surgery. However, one recent study indicated that this “IOP reduction” was not associated with preventing disease progression [1]. This may be due to biomechanical changes in the eye following cataract surgery [2]. Indeed, the biomechanical properties of the eye are also important in the disease because they affect IOP measurement [3,4]. Moreover, they play an important role in the pathogenesis of glaucoma [5]. Due to advancements in biometric techniques in recent decades, the biomechanical features of an eye can be measured directly. Corneal Visualization Scheimpflug Technology (Corvis ST; Oculus GmbH, Wetzlar, Germany) visualizes and records corneal deformation through the application of an air jet using an ultra-high-speed Scheimpflug camera in detail and provides data on biomechanical parameters. These Corvis ST parameters are associated with the severity [6] and visual field progression [7] of glaucoma.

We recently reported a considerable difference in the postoperative changes in biomechanical corneal properties between LOT combined with cataract surgery and solo cataract surgery. However, a more detailed investigation could not be conducted because LOT was combined with cataract surgery, and preoperative IOP was not matched between the two groups [8]. Thus, in this study, we compare the changes in corneal stiffness and maximum corneal deformation measured with Corvis ST between solo LOT and solo cataract surgeries, where background factors, such as preoperative IOP, age, and axial length, are matched.

## 2. Materials and Methods

The Research Ethics Committee of Seirei Hamamatsu General Hospital approved this retrospective study (#4027, approved on 3 August 2022) that was conducted following the tenets of the Declaration of Helsinki. All participants signed a written informed consent form permitting us to store and use their clinical information in the hospital database for research.

### 2.1. Participants

This study included glaucomatous patients who received solo LOT surgery and non-glaucomatous eyes that received solo cataract surgery. To avoid the interference of possible effects of these variables on the cornea’s biomechanical properties, preoperative IOP, age, and axial length were matched between the two groups. The inclusion criteria for the LOT group were as follows: (1) wide open angle with gonioscopy; (2) typical glaucomatous changes in the optic nerve head (e.g., rim notch with a rim width ≤ 0.1 disk diameter, a vertical cup-to-disk ratio > 0.7, or a retinal nerve fiber layer defect); and (3) glaucomatous VF defects compatible with the optic nerve head changes meeting the Anderson–Patella criteria [9] on two consecutive examinations. In the present study, we only included those with a preoperative IOP < 22 mmHg, so that a group matched with the cataract group could be created.

The solo cataract group included participants without any abnormal eye-related findings, except for cataracts on biomicroscopy, gonioscopy, and fundoscopy. Those with any history of ocular diseases, such as diabetic retinopathy or age-related macular degeneration, were excluded.

The exclusion criteria for the two groups excluded patients with any corneal abnormality that could affect the Corvis ST measurement, such as keratoconus, and contact lens wearers. Also, patients with conditions such as neurological disorders, cranial trauma, or neoplasms, as well as patients who used chloroquine medication that could result in the development of a VF loss, were excluded.

### 2.2. Surgical Technique

All procedures were performed at the Seirei Hamamatsu General Hospital between June 2020 and March 2023 by a single surgeon (R.A.). All surgeries were conducted under topical anesthesia. In the cataract group, a clear corneal incision of 2.4 mm in length was made and phacoemulsification with intraocular lens implantation in the posterior chamber was performed. Postoperatively, patients could receive topical anti-inflammatory, topical steroid, and topical antibiotic medications for up to 3 months, 6 weeks, and 6 weeks, respectively. In the LOT group, a trabeculotomy microhook was used to incise the trabecular meshwork through two quadrants. Postoperatively, the patients could receive topical steroids, topical antibiotics, and topical pilocarpine for up to 6 weeks, 6 weeks, and 6 months, respectively. In the LOT group, all antiglaucoma medications prescribed preoperatively were discontinued postoperatively; however, they were resumed at the discretion of the attending physician.

### 2.3. Clinical Data Acquisition

We collected background demographic data at baseline, such as preoperative IOP, age, sex, and axial length, from the medical charts. Axial length was measured preoperatively using the IOL Master ver. 5.02 (Carl Zeiss Meditec, Dublin, CA, USA). Corvis ST and Goldmann applanation tonometer (GAT) IOP measurements were performed preoperatively and 6 months postoperatively. Corvis ST was measured randomly at 15 min intervals. Eye drop score accounted for 1 point for a component of topical antiglaucomatous medication or a tablet of oral acetazolamide.

### 2.4. Corvis ST

The principles of Corvis ST measurements have been described in detail in other papers [8,10]. In brief, a high-speed Scheimpflug camera records 140 images of corneal deformation for 30 ms after the injection of an air pulse emitted from the device. Subsequently, the cornea experiences the first applanation during inward corneal movement (A1), at the highest concavity (HC), when the cornea is most significantly depressed, and the second applanation during outward corneal movement (A2). Using the recorded images, the Corvis ST device yields various parameters, such as corneal stiffness or deformation at the HC. In this study, all parameters were calculated using the latest version of the Corvis ST software (version 1.6r2223). More intuitive Corvis ST parameters were also calculated from raw parameters. For instance, “stiffness parameter A1” (SP-A1) and the “stress–strain index” (SSI) were also obtained as Corvis ST parameters related to corneal stiffness. SP-A1 is the ratio of the loading on the cornea to its deformation at the first applanation, calculated as follows:

$$\mathrm{SP}\text{-}A1=\frac{adjAP1-bIOP}{A1DefAmp}$$

where adjAP1 is the adjusted air pressure on the cornea at the first applanation, bIOP is the Corvis ST-derived biomechanical IOP, and A1DefAmp represents corneal apex displacement at the first applanation. A higher SP-A1 value indicates a stiffer cornea, as more loading is required to flatten the cornea [11]. Thus, SP-A1 shows a secant elastic modulus at a single state of the cornea, and the nonlinear relationship between IOP, corneal morphology, and elastic properties of the cornea was excluded in the formula [12], enabling the independence of SP-A1 on IOP and corneal geometry (e.g., CCT). In contrast, the SSI measures the nonlinearity of the stress–strain relationship of the eye; the SSI was developed using finite-element methods to represent corneal stiffness as materially independent from the IOP and corneal geometry [13]. The SSI is indicated on a normalized scale, where the average value for 50-year-old eyes is 1. Higher SSI values indicate stiffer and less deformable corneas. PD is the distance between nondeformed peaks at the HC, and DefAmpMax is the maximum displacement of the corneal apex compared to the baseline.

Each Corvis ST parameter was measured thrice, and the mean values were used for the analysis. Only reliable Corvis ST measurements were used, as suggested by the “OK” quality indicator displayed on the instrument monitor.

### 2.5. Statistical Analysis

The mean values of baseline clinical factors and Corvis ST parameters were compared between the LOT and cataract groups with linear mixed models, where the random effect was the patient, because of the inclusion of either one or two eyes. In addition, changes in SP-A1, SSI, PD, and DefAmpMAx between pre- and postoperative values (dSP-A1, dSSI, dPD, and dDefAmpMAx, respectively) were calculated, and their association with the GAT IOP change (dGAT IOP) was investigated using the linear mixed model. The marginal R^2^ value for the linear mixed model was calculated based on the method reported by Nakagawa et al. [14].

We used the statistical programming language R 4.4.1 (The R Foundation for Statistical Computing, Vienna, Austria) for the data processing and analyses.

## 3. Results

We included 37 eyes from 31 patients with glaucoma who underwent LOT and 37 eyes from 26 patients who underwent solo cataract surgery. In the LOT group, 30 eyes had primary open-angle glaucoma, 2 eyes had exfoliation glaucoma, 2 eyes had chronic angle closure glaucoma, 2 eyes had secondary open-angle glaucoma, and 1 eye had developmental glaucoma. Moreover, 16 eyes were phakic and the remaining 21 eyes were pseudophakic. The mean and SD of the mean deviation (MD) value measured with the Humphrey Field Analyzer (Carl Zeiss Meditec, Dublin, CA, USA) were −12.7 and 7.0 dB in the remaining 29 eyes. Such measurements were not conducted in the remaining eight eyes for various reasons, such as poor vision.

**Figure 1:**
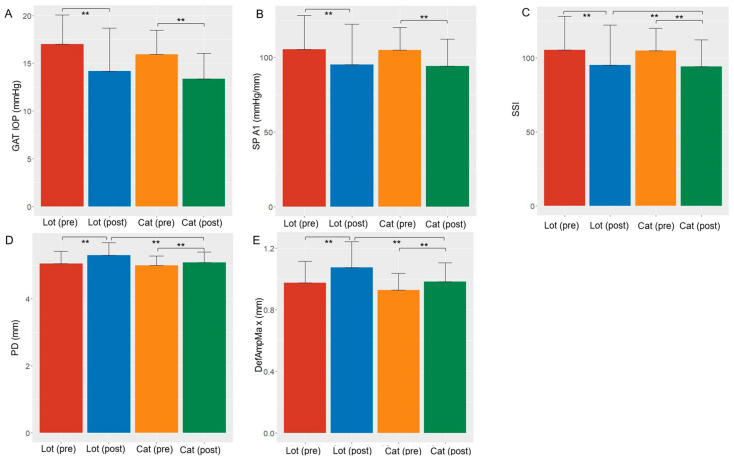
Comparisons of each variable between pre- and postoperative values and between each group: (**A**) GAT IOP, (**B**) SP-A1, (**C**) SSI, (**D**) PD, (**E**) DefAmpMax. GAT, Goldmann applanation tonometry; IOP, intraocular pressure; SP-A1, stiffness parameter upon first applanation; SSI, stress–strain index; PD, peak distance; DefAmpMax, maximum deformation amplitude. **: *p* < 0.01.

Table 1 and Figure 1 show the preoperative basic characteristics in each group. Preoperatively, the age, axial length, CCT, and GAT IOP score were similar between the two groups (all *p* > 0.05, linear mixed model). Additionally, PD, DefAmpMax, SP-A1, and SSI values were not significantly different between the two groups (*p* > 0.05, linear mixed model, adjusted for age, AL, and GAT IOP).

Table 1 and Figure 1 show the comparisons of each variable’s pre- and postoperative values within each group. GAT IOP was not significantly different when comparing the pre- and postoperative scores in both groups (all *p* > 0.05, linear mixed model). The pre- and postoperative eye drop scores were not significantly different within the LOT group.

Table 1 and Figure 1 also show the postoperative characteristics in each group. The GAT IOP was not significantly different between the two groups. In contrast, the LOT group had significantly larger PD and DefAmpMax values and a significantly smaller SSI compared with the cataract group (*p* < 0.001, linear mixed model, adjusted for age, AL, and GAT IOP). SP-A1 was not significantly different between both groups (*p* = 0.29).

Table 2 shows the associations between dSP-A1, dSSI, dPD, and dDefAmpMAx and dGAT IOP. All associations were significant in the LOT group (*p* < 0.001, linear mixed model), and this was similar in the Cat group, except for dPD. However, the Cat group’s mR^2^ values were much lower compared with those in the LOT group.

## 4. Discussion

Our study examined Corvis ST-measured postoperative changes in the corneal biomechanical properties in age-, AL-, and IOP-matched patients, namely in 37 glaucomatous eyes that received solo LOT and 37 eyes without glaucoma that received solo cataract surgery. Consequently, both groups showed a postoperative decrease in IOP. In addition, we found decreased corneal stiffness (as suggested by decreased SP-A1 and SSI) and corneal deformation (as suggested by increased PD and DefAmpMax) after LOT surgery, whereas only SP-A1 and DefAMpMax were changed in the Cat group. The SSI, PD, and DefAmpMax values showed a significantly greater change in the LOT group than in the Cat group.

Cataract surgery can reduce IOP by 3.4–1.5 mmHg [15,16,17,18]. Our findings showed a reduction in IOP by 2.50 mmHg in the cataract group (Table 1 and Figure 1). The IOP reduction following cataract surgery may be explained by several mechanisms. First, the posterior traction on the scleral spur by the anterior lens zonules becomes weak as the lens thickens, which results in a decrease in the outflow facility [18,19]. However, cataract surgery would resolve this condition. Particularly in eyes with a narrow angle, the reduction in lens volume results in angle widening and facilitates aqueous outflow [15]. Second, the outflow facility is increased due to the metalloproteinase production induced by the stress from high IOP or ultrasonic vibrations during phacoemulsification or the remodeling of the trabecular endothelium [20,21]. In addition, the cornea being more deformable would be another reason for “true” IOP reduction [4,22,23].

Previous studies have shown that IOP was reduced between 5 and 19.9 mmHg after LOT [24]. In the current study, the IOP reduction was 2.8 mmHg on average (Table 1 and Figure 1). This difference could be attributed to the difference in preoperative IOP values. Previous studies have shown that postoperative IOP after LOT is largely dependent on the preoperative IOP [24]. In contrast, our study only included those with a preoperative IOP < 22 mmHg in the analysis, resulting in the creation of a group matched with the cataract group.

Despite similar pre- and postoperative GAT IOP and preoperative Corvis parameters between both groups, the Corvis parameters were significantly different postoperatively (Table 1 and Figure 1). First, the SSI was reduced postoperatively in both groups, but it was greatly reduced significantly in the LOT group compared with the cataract group. The SSI is a parameter that was developed based on the findings obtained in a finite-element model experiment wherein biomechanical eye responses were analyzed at different IOP levels, and it is a parameter of corneal stiffness arising from material status, which is independent of IOP [13]. This implies that the reduced SSI in the cataract group could be attributed to the effect of the incision during the cataract surgery, whereas the incision in LOT surgery was very small, and its effect on corneal stiffness would be negligible. In eyes with glaucoma, the trabecular meshwork is stiff and resistant to external stress [25]. LOT removes this resistance, and even the pectinate ligament is incised. Our findings indicated that the effect of the incision in the trabecular meshwork and the pectinate ligament on SSI was significantly larger than that of the incision in cataract surgery. Although SP-A1 was significantly lower postoperatively than preoperatively in both groups, SP-A1 did not differ postoperatively between both groups. SP-A1 is dependent on the IOP; however, this cannot explain this finding because the pre- and postoperative IOP values were not significantly different between both groups (Table 1 and Figure 1). This result would indicate that the effects of the incision in cataract surgery and the pectinate ligament in LOT surgery on corneal stiffness are almost identical at the A1 time point (no significant difference in SP-A1). However, the latter was significantly larger after that time point (significant difference in SSI). The cornea being more deformable at the HC, as suggested by the larger PD and DefAmpMax values in the LOT group compared to the cataract group, would support this. Similarly, the changes in PD, DefAmpMax, SP-A1, and SSI were more closely related to the change in IOP in the LOT group than they were in the cataract group (Table 2).

Previous studies have suggested that IOP decreases after cataract surgery; however, the rate of visual field progression is not altered after this surgery [26,27]. Furthermore, in Kim et al.’s research, it was reported that visual field progression is actually even accelerated after cataract surgery compared to its preoperative level [1]. The mechanism for this result is unclear; however, a postoperative reduction in the measured IOP may not entirely be a true IOP reduction but may also occur due to postoperative biomechanical changes, such as corneal softening [2]. The cornea is similarly soft after LOT, but the biomechanical properties are also largely changed with this surgery.

Antiglaucoma medications have significant influences on not only IOP but also other biomechanical properties of the ocular tissue, such as corneal thinning [28,29,30] and softening [31,32]. We discontinued all topical antiglaucoma medications postoperatively, including prostaglandin analogs, but they were resumed at the discretion of the attending physician in the postoperative follow-up period. Thus, the eye drop score was not significantly different before and after LOT surgery, implying that this effect is only negligible.

The present study has limitations. The sample size was relatively small, and further validation of the current results in a larger dataset is required. Another limitation is the retrospective nature of this study: this study’s data are limited to a period of 6 months after the operation. Extended follow-up evaluation would be advantageous in comprehending the enduring biomechanical alterations and their consequences on visual outcomes and the advancement of diseases. Finally, although this study considered the effects of antiglaucoma drugs, their possible influence on corneal biomechanics could not be not thoroughly investigated because of the relatively small sample size. Thus, our findings should be considered as initial findings that necessitate additional ongoing research in order to validate the conclusions. Moreover, eyes with relatively low IOP were selected in the LOT group. This consideration was needed in our selection of glaucoma patients, because biomechanical corneal properties are closely associated with IOP, and postoperative changes in the biomechanical corneal properties cannot be compared between the two surgeries unless the pre- and postoperative IOP values are matched.

In conclusion, the results of the present study suggest that the cornea becomes softer and more deformable after LOT and cataract surgeries, but this effect was more pronounced in the LOT group.

## Figures and Tables

**Table 1:** Basic characteristics and biomechanical parameters at baseline.

Parameters	LOT Group		Cat Group		*p*-Value *	*p*-Value **	*p*-Value †	*p*-Value ††
	Pre	Post	Pre	Post				
Age (years)	75.3 ± 8.2		74.5 ± 7.7		-	-	0.71	-
	(52–88)		(60–90)					
Axial length (mm)	25.2 ± 2.2		24.0 ± 2.4		-	-	0.19	-
	[21.4–30.9]		[21.2–33.1]					
Eye drop score	2.4 ± 1.6	2.6 ± 1.2	-	-	-	-	-	-
	[0 to 5]	[0 to 5]						
GAT IOP (mmHg)	17.0 ± 3.1	14.2 ± 4.5	15.9 ± 2.5	13.4 ± 2.7	<0.001	<0.001	0.071	0.55
	[8.0–21.0]	[6.0–29.0]	[8.0–21.0]	[9.0–23.0]				
CCT (µm)	528.5 ± 37.0	526.6 ± 37.6	541.8 ± 27.0	544.2 ± 28.3	0.32	0.33	0.063	0.023
	[456.0–608.0]	[452.0–606.0]	[490.0–590.0]	[490.0–606.0]				
Peak Distance (mm)	5.1 ± 0.36	5.3 ± 0.38	5.0 ± 0.29	5.1 ± 0.32	<0.001	0.24	0.62	<0.001
	[4.2–5.8]	[4.5–6.1]	[4.1–5.7]	[4.1–5.8]				
Maximum deflection amplitude (mm)	0.97 ± 0.14	1.08 ± 0.17	0.93 ± 0.11	0.98 ± 0.12	<0.001	<0.001	0.31	<0.001
	[0.72–1.3]	[0.76–1.4]	[0.69–1.2]	[0.67–1.2]				
SP-A1 (mmHg/mm)	105.4 ± 22.7	95.3 ± 26.9	105.0 ± 15.0	94.2 ± 17.9	<0.001	0.0015	0.98	0.29
	[67.5–150.4]	[55.4–153.5]	[82.0–137.1]	[64.3–136.4]				
SSI	1.19 ± 0.29	1.04 ± 0.24	1.28 ± 0.24	1.21 ± 0.25	<0.001	0.068	0.55	<0.001
	[0.66–1.87]	[0.64–1.60]	[0.77–1.79]	[0.73–1.73]				

LOT, solo trabeculotomy; Cat, solo cataract surgery; CCT, central corneal thickness; GAT, Goldmann applanation tonometry; SP-A1, stiffness parameter upon first applanation; SSI, stress–strain index. *p*-values according to linear mixed model: * between pre- and postoperative values (LOT group); ** between pre- and postoperative values (Cat group); †, between LOT and Cat groups (pre operation); ††, between LOT and Cat groups (post operation).

**Table 2:** Associations between Corvis ST parameters and GAT IOP changes.

	LOT Group				Cat Group			
	Partial Regression Coefficient	SE	*p*-Value	mR^2^	Partial Regression Coefficient	SE	*p*-Value	mR^2^
Change in peak distance (mm)	−0.044	0.0086	<0.001	0.42	−0.023	0.012	0.065	0.11
Change in maximum deflection amplitude (mm)	−0.022	0.0035	<0.001	0.52	−0.018	0.0048	<0.001	0.31
Change in SP-A1 (mmHg/mm)	2.33	0.46	<0.001	0.39	2.72	0.87	0.0039	0.25
Change in SSI	0.029	0.0069	<0.001	0.32	0.035	0.011	0.0035	0.25

LOT, solo trabeculotomy; Cat, solo cataract surgery; SE, standard error; GAT, Goldmann applanation tonometry; SP-A1, stiffness parameter upon first applanation; SSI, stress–strain index.

## Data Availability

All data can be made available if a request is made to the corresponding author.
